# Proliferation and Activation of Osterix‐Lineage Cells Contribute to Loading‐Induced Periosteal Bone Formation in Mice

**DOI:** 10.1002/jbm4.10227

**Published:** 2019-09-11

**Authors:** Heather M Zannit, Matthew J Silva

**Affiliations:** ^1^ Department of Orthopaedic Surgery and Department of Biomedical Engineering Washington University St Louis MO 63110 USA

**Keywords:** LINEAGE TRACING, MECHANICAL LOADING, OSTEOBLAST, OSTERIX, PERIOSTEUM

## Abstract

Mechanical loading stimulates bone formation. Bone‐lining‐cell activation and cell proliferation have been implicated in this process. However, the origin of osteoblasts that form bone following mechanical stimulation remains unknown. Our objective was to identity the contributions of activation, differentiation, and proliferation of osteoblast lineage cells to loading‐induced periosteal bone formation. Tamoxifen‐inducible Osx‐Cre‐ERT2;Ai9/TdTomato reporter mice (male and female) were aged to young adult (5 months) and middle age (12 months), and were administered tamoxifen for 5 consecutive days to label osterix‐lineage cells. Following a 3‐week clearance period, mice were subjected to five consecutive bouts of unilateral axial tibial compression. We first confirmed this protocol stimulated an increase in periosteal bone formation that was primarily lamellar apposition. Next, mice received 5‐bromo‐2′‐deoxyuridine (BrdU) in their drinking water daily to label proliferating cells; calcein was given to label active mineralizing surfaces. Tibias were harvested after the fifth loading day and processed for frozen undecalcified histology. The middiaphyseal periosteal surface in the region of peak bone formation was analyzed. Histology revealed both nonloaded and loaded tibias were covered in osterix positive (Osx^+^) cells on the periosteal surface of both 5‐ and 12‐month‐old animals. There was a significant increase in the mineralizing surface (calcein^+^) covered with Osx^+^ cells in loaded versus control limbs. Furthermore, nearly all of the mineralizing surfaces (>95%) were lined with Osx^+^ cells. We also observed approximately 30% of Osx^+^ cells were also BrdU^+^, indicating they arose via proliferation. These results show that following mechanical loading, pre‐existing cells of the Osx lineage cover the vast majority of surfaces where there is active loading‐induced bone formation, and a portion of these cells proliferated in the 5‐day loading period. We conclude the initial anabolic response after mechanical loading is based on the activation and proliferation of Osx lineage cells, not the differentiation of progenitor cells. © 2019 The Authors. *JBMR Plus* published by Wiley Periodicals, Inc. on behalf of American Society for Bone and Mineral Research.

## Introduction

Mechanical loading is a potent anabolic stimulus that promotes bone formation.^1^, ^2^ After skeletal development, bone formation occurs when osteoblasts are either activated from lining cells on the bone surface or recruited through differentiation or proliferation. The relative contribution of these different mechanisms to osteoblasts that make bone after mechanical loading is not known.

Previous research has described activation and proliferation as contributing to osteoblasts after mechanical loading. In adult animals, bone surfaces of nonloaded limbs are covered with quiescent, flat bone‐lining cells,^3^, ^4^, ^5^, ^6^, ^7^ characterized by their condensed cell height, small cytoplasm, and spindle‐shaped nuclei.^5^, ^8^ As early as 2 days after loading, these lining cells can become activated, as demonstrated by an increase in cell number, cell height, and cell layers, and by the appearance of rounded nuclei.^3^, ^4^, ^5^, ^6^, ^8^, ^9^ Moreover, proliferation can occur 48 to 96 hours after a single bout of loading, contributing 30% to 40% of the bone cells on the endocortical surface after four‐point tibial bending.^9^ These earlier studies were limited by the tools that existed to characterize bone cells with simple histological techniques. The objective of the current study was to use modern lineage‐tracing methods to better define the origin of cells on the bone surface after anabolic mechanical loading, including those that arise via cell proliferation.

Recent studies have used inducible Cre reporter mice to track osteoblast lineage cells in skeletal development and fracture repair.^10^, ^11^, ^12^, ^13^, ^14^, ^15^ Osteoblasts are derived from mesenchymal progenitor cells, which differentiate into osteoprogenitors (or preosteoblasts), and then mature osteoblasts.^16^ The cells of the osteoblast lineage responsible for bone formation can be well‐visualized on the periosteal surface of the tibia. Directly on the bone surface are mature osteoblasts with a cuboidal morphology and flat bone‐lining cells. Covering the osteoblast surface is a fibrous membrane, known as the periosteum. Within the periosteum's two layers reside the preosteoblasts in the inner cambium layer and the mesenchymal progenitors in the outer fibrous layer appearing as elongated fibroblasts.^16^, ^17^, ^18^, ^19^ An important stage in this lineage is marked by the expression of osterix (Osx), a transcription factor that is downstream of Runx2 and is expressed as preosteoblasts become osteoblasts.^11^, ^20^ Osx^+^ cells have been seen in the periosteum and among trabecular osteoblasts, endocortical osteoblasts, periosteal osteoblasts, and osteocytes.^11^, ^21^, ^22^ In developing bones, about 22% of Osx^+^ cells were found to proliferate, but in 8‐ to 10‐week‐old mice less than 1% of Osx^+^ cells were found to proliferate.^11^, ^22^ Thus, Osx marks an osteoblast lineage cell that may re‐enter the cell cycle, albeit infrequently in the mature skeleton.

We utilized an Osx‐CreERT2‐inducible reporter mouse to pulse‐label cells expressing Osx prior to mechanical loading, and then chased this cell population to characterize how these cells contributed to periosteal bone formation. We used skeletally mature mice at 5 months (young adult) and 12 months (middle age), and subjected them to 5 days of axial tibial loading to induce periosteal bone formation. We asked several questions. What fraction of periosteal cells at sites of bone formation derives from pre‐existing Osx^+^ cells? How many of these cells, if any, arise via cell proliferation?

## Subjects and Methods

### Animal studies

This study was approved by the Washington University Institutional Animal Care and Use Committee (IACUC). Transgenic Osx‐Cre‐ERT2 mice^10^ (provided by Henry Kronenberg, Harvard University, Cambridge, MA, USA) that were maintained on a mixed background, and Ai9/tdTomato reporter mice^23^ (B6.Cg‐*Gt(ROSA)26Sor* ^*tm9(CAG‐tdTomato)Hze*^/J, #007909; The Jackson Laboratory, Bar Harbor, ME, USA) were used to generate inducible reporter mice. Experimental animals were Osx‐Cre‐ERT2^+/−^;Ai9^+/+^ (iOsx‐Ai9) that were administered tamoxifen. Control animals were either iOsx‐Ai9 without tamoxifen (*n* = 8), used to evaluate the periosteal surface for Cre leakiness and to ensure tamoxifen treatment did not alter the loading response (ie, calcein labeling), or Osx‐Cre‐ERT2^−/−^;Ai9^+/+^ (Ai9; *n* = 18) administered tamoxifen, used for Cre specificity and to evaluate any possible Cre side‐effects. One month prior to mechanical loading, adult male and female iOsx‐Ai9 mice were administered tamoxifen (TAM; Sigma‐Aldrich Corp., St. Louis, MO, USA) for 5 consecutive days (75 mg/kg/day i.p. injection) followed by a 3‐week clearance interval to allow for efficient labeling of pre‐existing Osx‐lineage cells and to avoid the short‐term effects of tamoxifen on bone formation.^24^, ^25^ Tamoxifen was only administered prior to mechanical loading and was not given continuously, allowing for a “pulse‐chase” lineage study. Preliminary studies confirmed that this dosing protocol had no influence on subsequent loading‐induced bone formation (Supplementary Fig. 1). Mice were group‐housed by gender under a standard 12‐hour light/dark cycle and given access to food and water *ad libitum*. Mice were euthanized by CO_2_ asphyxiation at designated time points.

### In vivo tibial compression

Adult male and female transgenic mice aged 5 and 12 months were anesthetized using 3% isoflurane and placed on a loading platform in a materials testing system (Electropuls 1000; Instron, Norwood, MA, USA). A preload (−0.5 N) was applied and the right tibias were cyclically loaded for 5 consecutive days (1200 cycles/d, 4‐Hz triangle waveform with 0.1‐sec rest‐insertion (−0.5 N) after each cycle) to induce cortical bone formation.^26^, ^27^ Because our primary goal was to study cell recruitment in the context of physiological loading‐induced bone formation, we selected peak force values empirically to induce a similar anabolic response of lamellar bone formation with minimal woven bone across age and sex groups. A range of force values were evaluated in preliminary studies and qualitative assessment based on dynamic histomorphometry was used to select the target forces (Supplementary Table 1). Accordingly, male 5‐ and 12‐month‐old mice were loaded to –11 N and –14 N, respectively, while female 5‐ and 12‐month‐old mice were loaded to –7 N and –8 N, respectively. Mice received buprenorphine (0.1 mg/kg s.c.) following each loading bout for analgesia, were returned to their cages to resume normal activity, and were monitored daily. The left tibias were not loaded and served as contralateral controls.

### Dynamic histomorphometry

**Figure 1:**
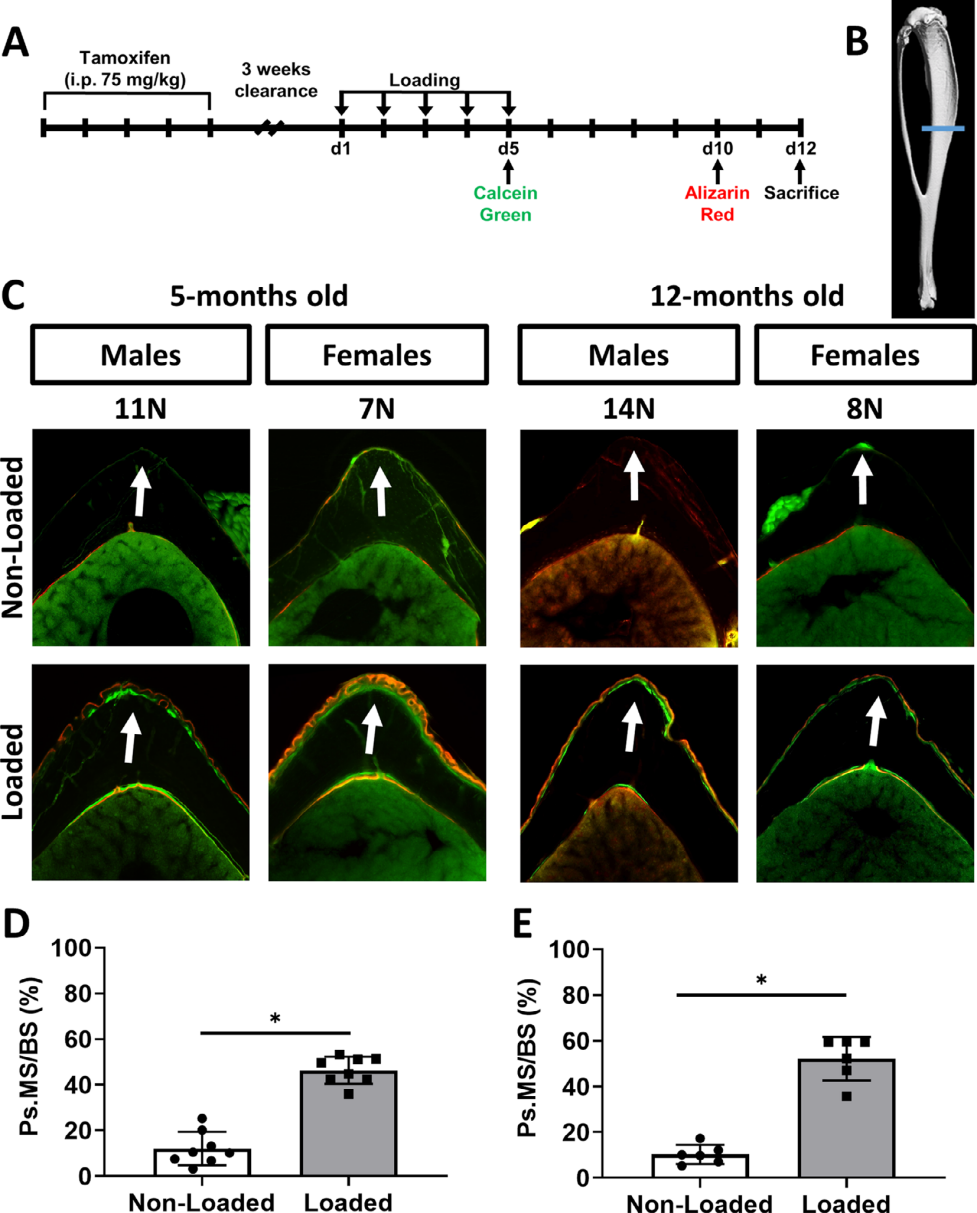
Mechanical loading‐induced bone formation occurs in tibias of 5‐ and 12‐month‐old iOsx‐Ai9 mice. (*A*) Cre activity was induced by five consecutive doses of tamoxifen (75 mg/kg) followed by a 3‐week clearance. Tibias were then loaded for 5 consecutive days; mineralizing surfaces were labeled with calcein and Alizarin on days 5 and 10, respectively. Mice were euthanized on day 12 for histological evaluation. (*B*) Transverse sections were cut 5‐mm proximal to the distal tibial–fibular junction. (*C*) Representative transverse sections of fluorochrome‐labeled nonloaded and loaded tibias illustrating an increase in periosteal (Ps) bone on the peak compressive surface (arrows). Analysis of the periosteal surface revealed that loaded tibias from (*D*) 5‐ and (*E*) 12‐month‐old mice had an increase in periosteal mineralizing surface (Ps.MS/BS). Data shown as mean ± SD. * Signifies *p* < .05 by Student's *t* test [5‐month‐old mice: *n* = 8 (males = 5, females = 3); 12‐month‐old mice: *n* = 6 (males = 3, females = 3)].

To first confirm an anabolic response to the loading protocol, a set of 5‐ and 12‐month‐old iOsx‐Ai9 mice (*n* = 8 and *n* = 6, respectively) were loaded for assessment of bone formation by dynamic histomorphometry. After loading on days 1 to 5, fluorochromes were injected i.p. on days 5 and 10 with calcein green (10 mg/kg; Sigma‐Aldrich) and Alizarin complexone (30 mg/kg; Sigma‐Aldrich), respectively. Mice were euthanized on day 12 (Fig. 1 *A*). Tibias were harvested and embedded undecalcified in plastic. Dynamic histomorphometric analysis was performed on 30‐μm‐thick transverse sections taken at sites of peak bone formation located 5‐mm proximal to the distal tibial–fibular junction (Fig. 1 *B*).^28^ Using commercial software (Osteo II; Bioquant Image Analysis Corp., Nashville, TN, USA), the periosteal mineralizing surface (Ps.MS/BS), the mineral apposition rate (Ps.MAR), and the bone‐formation rate (Ps.BFR/BS), where BS is the entire periosteal circumference, were determined as measures of loading‐induced bone formation. For samples with no double‐labeled surface, a minimum value of 0.3 μm/d was assigned for Ps.MAR and was used to calculate Ps.BFR/BS.^29^

### Histology for lineage tracing, bone formation, and cell proliferation

At the beginning of loading (day 1) until the end of the study (day 5), 5‐ and 12‐month‐old iOsx‐Ai9 experimental mice (*n* = 18 and *n* = 20, respectively) received 5‐bromo‐2′‐deoxyuridine (BrdU; Sigma‐Aldrich) in their drinking water daily (0.8 mg/mL, with 5% sucrose) to label all cells that arose by proliferation. On day 4 of loading, mice received an injection of calcein green (10 mg/kg) immediately after loading and again 6 to 8 hours later to label mineralizing surfaces (Fig. 3 *A*). On day 5, 2 to 3 hours following the last bout of loading, mice were euthanized. Tibias were fixed in 4% paraformaldehyde for 36 hours, rinsed in PBS, infiltrated in 30% sucrose, and embedded in optimal cutting temperature compound (OCT compound, Tissue‐Tek; VWR, Leicestershire, UK). Intestines were harvested and placed in 10% NBF and processed for paraffin sectioning as an internal control for BrdU incorporation. Longitudinal 5‐um‐thick cryosections were cut to intersect the posterolateral surface of the tibia, which is the site of peak compressive strain in this model (Fig. 3 *B*).^28^, ^30^, ^31^ Using a modification of the Kawamoto tape‐transfer technique (Section‐Lab Co. Ltd, Hiroshima, Japan), sections were mounted on glass slides using a 1% chitosan adhesive.^32^, ^33^ Frozen sections were mounted with 4,6‐diamidino‐2‐phenylindole (DAPI; #17985–50; Electron Microscopy Sciences, Hatfield, PA, USA), and the whole tibia was imaged using a Zeiss Axio Scan.Z1 slide scanner (Carl Zeiss, Thornwood, NY, USA) with DAPI, red fluorescent protein, and green fluorescent protein (GFP) filters to visualize nuclear, Cre‐activated tdTomato, and calcein green expression, respectively. Slides were then de‐cover‐slipped in PBS for further histological staining.

#### BrdU staining

To assess cell proliferation, frozen bone sections were rehydrated in PBS, and paraffin ileum sections were deparaffinized and rehydrated in graded alcohol. A 1% trypsin in PBS solution was placed on the tissue for 10 min at 37°C. Slides were rinsed in PBS followed by 4 N HCl for 30 min at room temperature. Slides were rinsed in PBS then blocked in mouse‐on‐mouse blocking reagent (Vector Labs, Burlingame, CA, USA) for 10 min. A biotinylated monoclonal BrdU antibody (B35138, diluted 1:50 in PBS; Thermo Fisher Scientific, Waltham, MA, USA) was placed on the tissue and incubated overnight at 4°C. Frozen sections were brought to room temperature, rinsed in 1X PBS, and incubated with a Alexa Fluor 488 Streptavidin conjugate (S11223, diluted 1:100 in PBS; Thermo Fisher Scientific) for 1.5 hours. Sections were then rinsed and cover‐slipped with Prolong Diamond Antifade with DAPI (P36966; Thermo Fisher Scientific). Following antibody treatment, paraffin ileum sections proceeded with a HRP‐streptavidin and DAB substrate reaction and were counterstained with hematoxylin. For paraffin histology, bright‐field BrdU images were obtained using the Zeiss Axio Imager Z2 microscope and a 20× objective. For frozen histology, fluorescent images were obtained along the periosteal compressive surface on the Zeiss Axio Imager Z2 with the GFP filter used to detect BrdU Alexa 488.

#### Histology analysis

A 2‐mm region of interest (ROI) was designated on the periosteal compressive surface centered at 5‐mm distal to the tibial plateau. This region is where we and others have recorded peak surface strains induced by loading, and subsequent periosteal and endocortical bone formation.^27^, ^28^ This ROI covers the same area measured in our transverse assessments of load‐induced bone formation by dynamic histomorphometry (Fig. 1). This ROI was found on the whole‐tibia scan and on the BrdU images. The two images were then overlaid using Zeiss Zen image‐processing tools to have a single image showing tdTomato, calcein, DAPI, and BrdU signals. The cancellous bone beneath the growth plate of the tibia metaphysis was examined to ensure calcein was incorporated into the bone. The endocortical surface was excluded from analysis because of technical histological issues in viewing cells along the entire surface within our ROI. Tibial sections were excluded from analysis because of technical errors in tissue processing (*n* = 10), absent BrdU staining in bone marrow (*n* = 8), and periosteal tissue damage following BrdU staining (*n* = 4). BIOQUANT was used to characterize the cells within the first layer of the periosteum on the bone surface within the ROI to determine: (1) the percentage of the bone surface that is tdTomato^+^, calcein^+^, DAPI^+^, BrdU^+^, and all combinations thereof; and (2) the number of DAPI^+^, tdTomato^+^, and BrdU^+^ cells.

### Statistics

No gender effect was found from a two‐way repeated measures ANOVA (with gender as a between factor and loading as a repeated factor); therefore, data from males and females were pooled for both dynamic histomorphometry and frozen histology. A paired Student's *t* test was used to compare bone formation indices between loaded and nonloaded tibias to determine a mechanical loading effect. Statistical significance was considered as *p* < .05, with trends noted for 0.05 < *p* < .10. Analysis was performed with Prism 8.0 (GraphPad, La Jolla, CA, USA). Age groups were considered separately and not directly compared. Individual data points and the mean ± SD are plotted.

## Results

### Mechanical loading induces periosteal bone formation in 5‐ and 12‐ month‐old iOsx‐Ai9 mice

Cre recombination was induced by 1 week of tamoxifen dosing in mice beginning 4 weeks prior to mechanical loading, which was performed at 5 and 12 months of age for 5 consecutive days (Fig. 1 *A*). Dynamic histomorphometry analysis revealed that in both 5‐ and 12‐month‐old male and female mice, there was a significant increase in periosteal mineralizing surface (Ps.MS/BS) in loaded compared with nonloaded tibias (Fig. 1 *C–E*). Nonloaded tibias from 5‐ and 12‐month‐old mice showed marginal periosteal bone formation, whereas loaded tibias had single or double labels on greater than 50% of the periosteal surface (Table 1). A significant increase was also found in Ps.MAR and Ps.BFR/BS in 5‐ and 12‐month‐old mice (Table 1). Thus, iOsx‐Ai9 mice displayed an anabolic response to axial tibial loading, as anticipated.

**Table 1:** Periosteal Bone Formation Indices

Outcomes	5 Months		12 Months	
	Nonloaded	Loaded	Nonloaded	Loaded
Ps.MS/BS (%)	12.1 ± 7.3	46.4 ± 5.9^a^	10.3 ± 4.2	52.2 ± 9.6^a^
Ps.MAR (μm/d)	0.37 ± 0.21	2.55 ± 0.53^a^	0.42 ± 0.30	2.42 ± 0.45^a^
Ps.BFR/BS (μm^3^/μm^2^/d)	0.04 ± 0.03	1.19 ± 0.29^a^	0.04 ± 0.03	1.26 ± 0.35^a^

Values are mean ± SD; 5‐month‐old mice: *n* = 8; males = 5, females = 3.

12‐month‐old mice: *n* = 6; males = 3, females = 3. A minimum value of 0.3 μm/d was assigned for MAR of nonloaded samples.^29^

Ps.MS/BS = periosteal mineralizing surface; Ps.MAR = periosteal mineral apposition rate; Ps.BFR/BS = periosteal bone‐formation rate.

a Signifies *p* < .05 nonloaded versus loaded.

### Periosteal Osx‐tdTomato expression in the tibia of iOsx‐Ai9 mice

**Figure 2:**
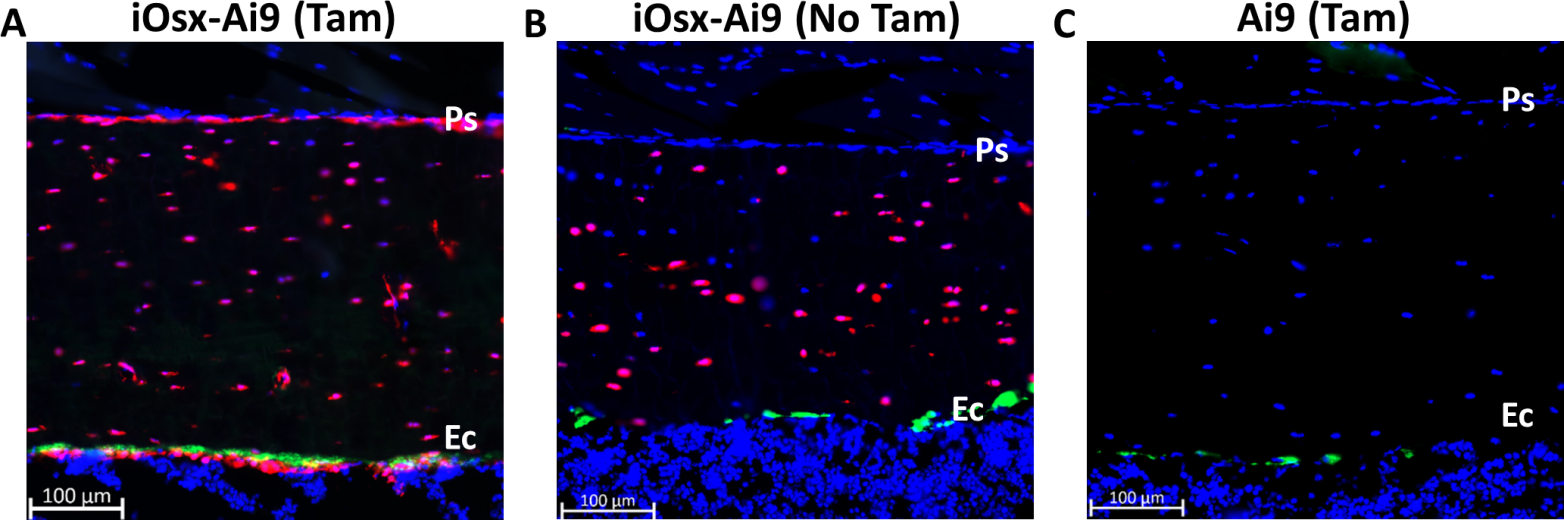
(*A*) TdTomato expression present in the osteocytes, periosteal, and endocortical cells of iOsx‐Ai9 mice that were administered tamoxifen (TAM). (*B*) TdTomato signal is seen in little to none of the periosteal surface in iOsx‐Ai9 mice in the absence of tamoxifen, but signal is present in approximately 50% of osteocytes. (*C*) No cells expressed tdTomato in Ai9 mice administered tamoxifen. Representative images of (*A*) *n* = 19 (males = 10, females = 9), (*B*) *n* = 5 (males = 2, females = 3), and (*C*) *n* = 17 (males = 10, females = 7). tdTomato = red; calcein = green; 4,6‐diamidino‐2‐phenylindole (DAPI) = blue; Ps = periosteum; Ec = endocortical.

Following a 3‐week clearance after tamoxifen administration, tdTomato expression was present in the osteoblasts on the periosteal and endocortical surface, as well as in the osteocytes of iOsx‐Ai9 mice, but absent in the marrow and muscle (Fig. 2 *A*). In the absence of tamoxifen, tdTomato expression was present in less than 5% of the osteoblasts on the periosteal surface (Fig. 2 *B*, Supplementary Fig. 2 *A, B*) and approximately 50% of the osteocytes. In Cre‐negative Ai9 mice, no tdTomato expression was present following tamoxifen administration (Fig. 2 *C*). Thus, tamoxifen induces labeling of Osx^+^ cells on the periosteum of adult iOsx‐Ai9 mice.

### Pre‐existing Osx‐lineage cells line the periosteal surface of nonloaded and loaded tibias in 5‐ and 12‐month‐old iOsx‐Ai9 mice

**Figure 3:**
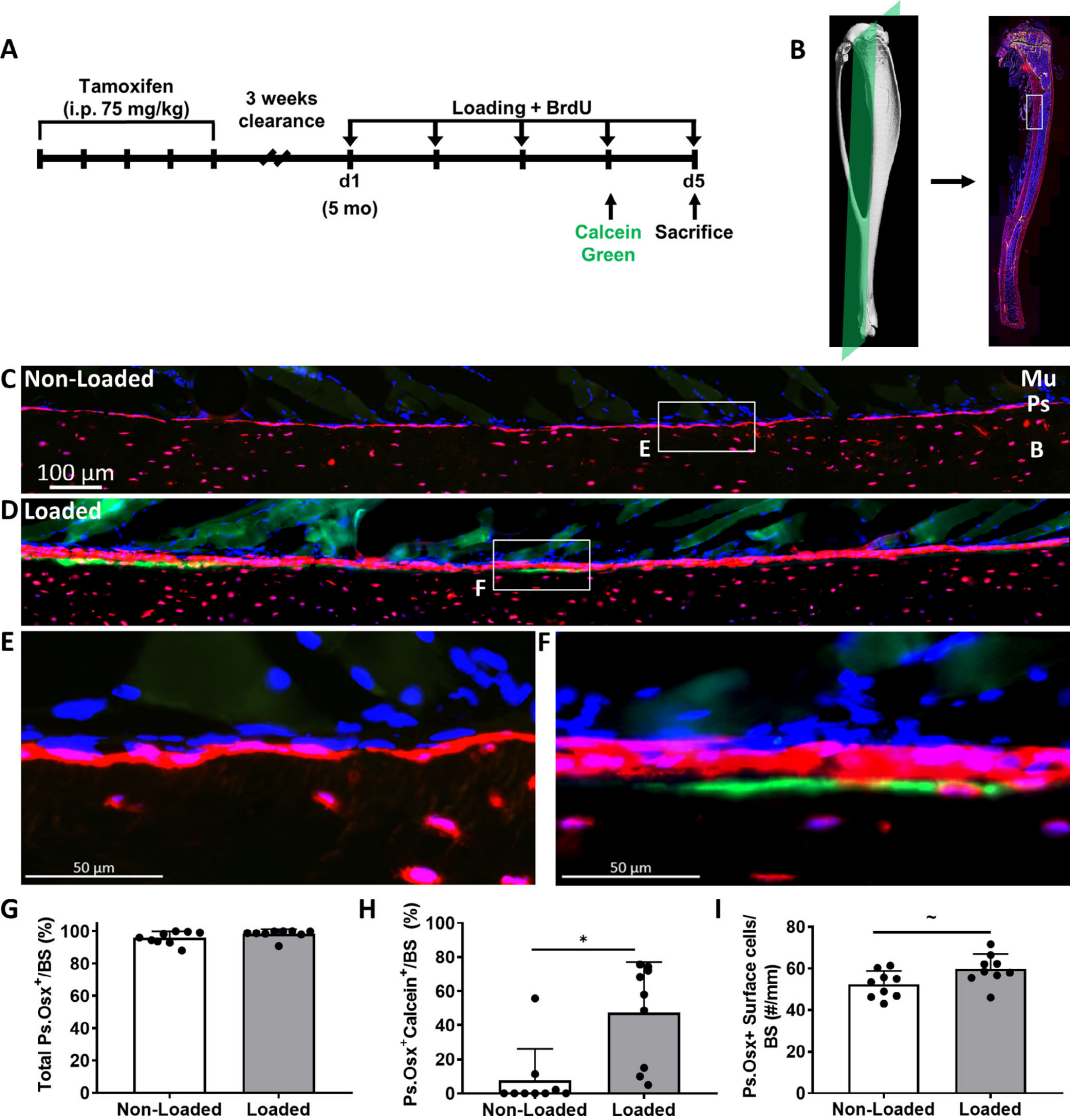
(*A*) Cre activity was induced by five consecutive doses of tamoxifen followed by a 3‐week clearance. Five‐month‐old mice had their right tibias loaded for 5 consecutive days. Mice received 5‐bromo‐2′‐deoxyuridine (BrdU) in their drinking water at the start of loading, calcein green on day 4, and were euthanized on day 5. (*B*) Longitudinal cryosections were cut to intersect the posterolateral surface of the tibia. (*C, D*) Representative images of the region of interest from nonloaded and loaded tibias. (*E, F*) 40× Magnifications from nonloaded and loaded tibias. (*G*) Total Ps.Osx^+^ (periosteum osterix positive) surface of nonloaded and loaded tibias. (*H*) Increase in Ps.Osx^+^ calcein^+^ surface with mechanical loading. (*I*) A trend for an increase was found in the number of periosteal Osx^+^ cells on the bone surface with loading. Data shown as mean ± SD (*n* = 9; males = 4, females = 5). * Signifies *p* < .05 and ~ signifies 0.05 < *p* < 0.1 by Student's *t* test. tdTomato = red; calcein = green; 4,6‐diamidino‐2‐phenylindole (DAPI) = blue; Mu = muscle; Ps = periosteum; B = bone.

In 5‐month‐old iOsx‐Ai9 mice treated with tamoxifen (Fig. 3), the periosteal surfaces of both nonloaded and loaded tibias were covered in tdTomato^+^ cells (96% and 98%, respectively, *p* = .134; Fig. 3 *C–G*). Nonloaded tibias exhibited a single layer of tdTomato^+^ cells on the periosteal bone surface (Fig. 3 *C*, *E*), whereas in loaded tibias, there was often more than one layer of tdTomato^+^ cells (Fig. 3 *D*, *F*). A significant increase in periosteal mineralizing surface (Osx^+^Calcein^+^) was found in loaded compared with nonloaded controls (47% and 8%, respectively, *p* = .009; Fig. 3 *H*), consistent with an anabolic response to loading. Notably, the mineralizing surfaces were always covered by tdTomato^+^ cells. Control 5‐month‐old mice exhibited a similar anabolic response (Supplementary Fig. 2 *C*), indicating no tamoxifen effect (iOsx‐Ai9, no tamoxifen) and no Cre effect (Ai9, tamoxifen). A trend for a slightly greater number of Osx^+^ cells on the periosteal surface was found in loaded compared with nonloaded controls (*p* = .06; Fig. 3 *I*). Embedding osteocytes were observed in all loaded tibias analyzed (9/9), as indicated by the tdTomato^+^ cells located in line with the calcein or between the calcein label and the bone surface (Fig. 3 *F*).

**Figure 4:**
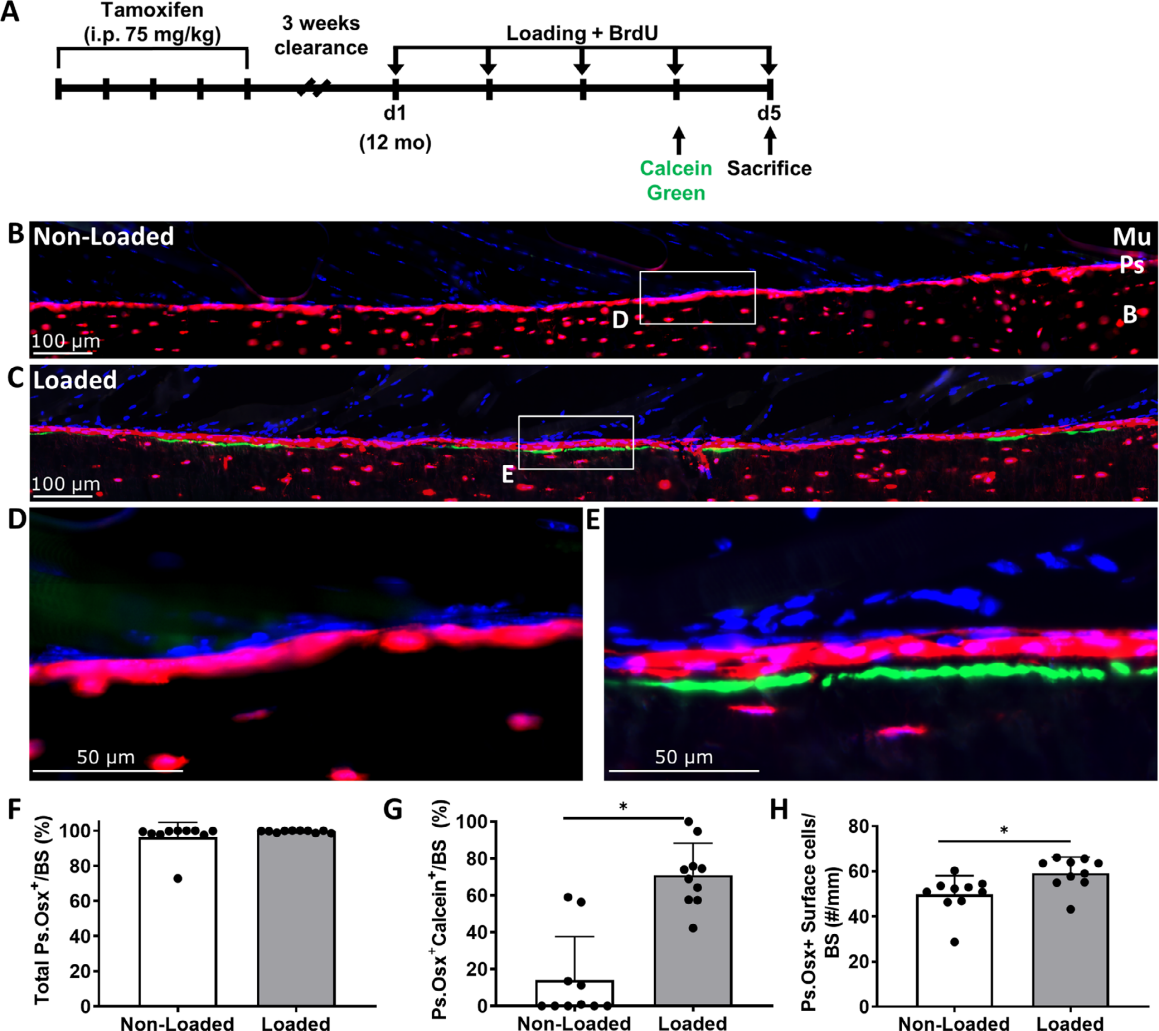
(*A*) Cre activity was induced by five consecutive doses of tamoxifen followed by a 3‐week clearance. Twelve‐month‐old mice had their right tibias loaded for 5 consecutive days. Mice received 5‐bromo‐2′‐deoxyuridine (BrdU) in their drinking water at the start of loading, calcein green on day 4, and were euthanized on day 5. (*B, C*) Representative images of the region of interest from nonloaded and loaded tibias. (*D, E*) 40× magnifications from nonloaded and loaded tibias. (*F*) Total Ps.Osx^+^ (periosteum osterix positive) surface of nonloaded and loaded tibias. (*G*) Increase in Ps.Osx^+^ calcein^+^ surface with mechanical loading. (*H*) A significant increase was found in the number of periosteal Osx^+^ cells on the bone surface with loading. Data shown as mean ± SD (*n* = 10; males = 6, females = 4). * Signifies *p* < .05 by Student's *t* test. tdTomato = red; calcein = green; 4,6‐diamidino‐2‐phenylindole (DAPI) = blue; Mu = muscle; Ps = periosteum; B = bone.

Findings in 12‐month‐old iOsx‐Ai9 mice (Fig. 4) were similar to the results at 5 months (Fig. 3). Both nonloaded and loaded tibias were covered in tdTomato^+^ cells (97% and 100%, respectively, *p* = .295; Fig. 4 *B–F*). The mineralizing surface (Osx^+^Calcein^+^) was significantly greater in loaded compared with nonloaded controls (71% and 14%, respectively, *p* < .0001; Fig. 4 *G*). Additionally, the mineralizing surface was 100% covered by tdTomato^+^ cells (Fig. 4 *E*). Control 12‐month‐old mice showed a similar anabolic response to loading (Supplementary Fig. 2 *D*), again indicating no tamoxifen effect (iOsx‐Ai9, no tamoxifen) and no Cre effect (Ai9, tamoxifen) on the loading response. A significant increase in the number of Osx^+^ cells on the periosteal surface was found in loaded compared with nonloaded controls (*p* = .023; Fig. 4 *H*). Finally, in 9 of 10 loaded tibias analyzed, we observed the presence of embedding osteocytes as indicated by the tdTomato^+^ cells located between the calcein label and the bone surface (Figs. 4 *C* and 6 *C, D*). Thus, in loaded tibias of 5‐ and 12‐month‐old iOsx‐Ai9 mice, all cells on the periosteal surface after 5 days of loading derive from pre‐existing Osx‐lineage cells.

### Mechanical loading stimulates proliferation of Osx‐lineage cells in 5‐ and 12‐month‐old iOsx‐Ai9 mice

**Figure 5:**
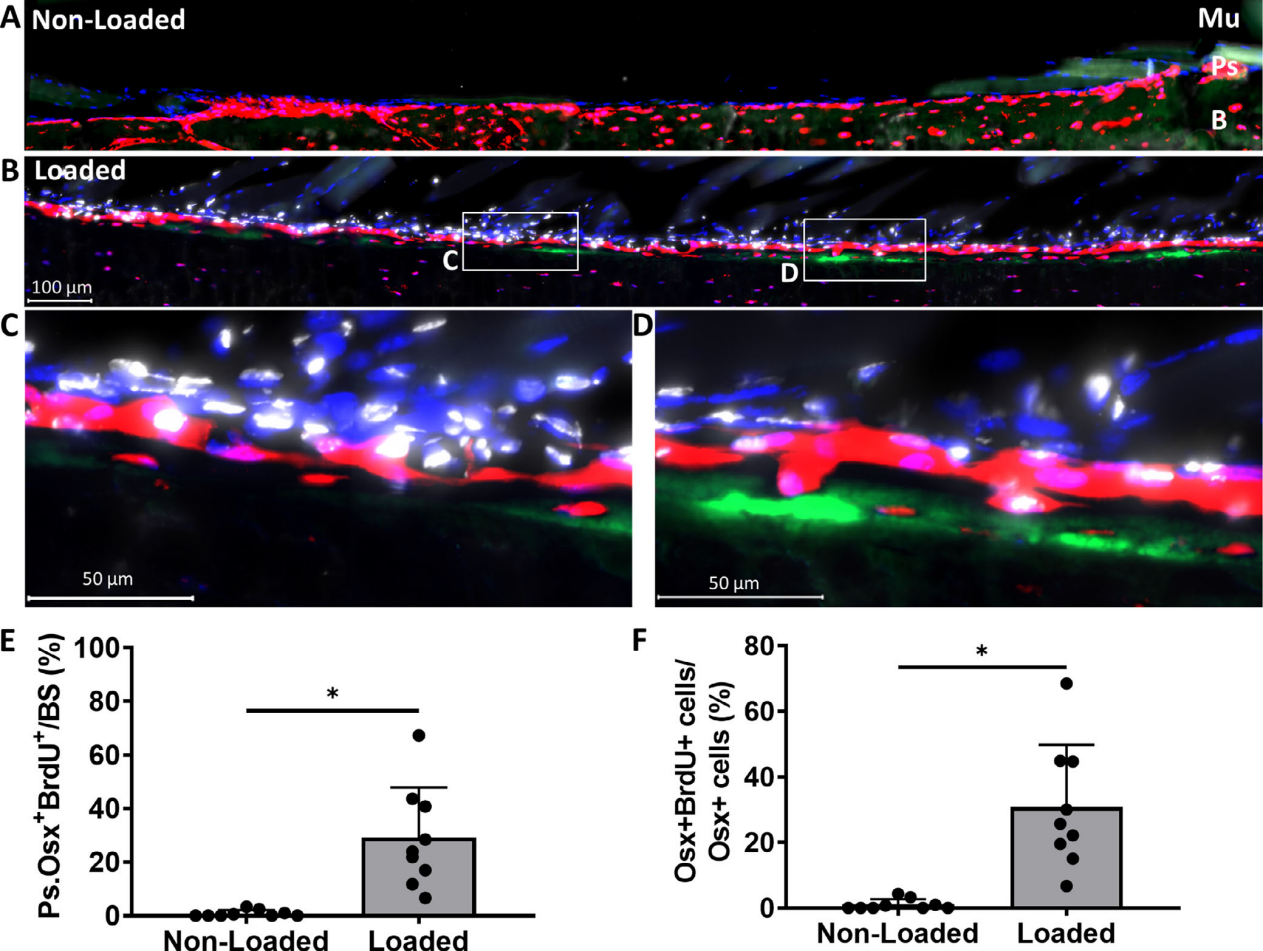
(*A, B*) Representative 5‐bromo‐2′‐deoxyuridine (BrdU) images of the region of interest from nonloaded and loaded tibias in 5‐month‐old mice. (*C, D*) 40× magnifications from loaded tibias. (*E*) Total Ps.Osx^+^ (periosteum osterix positive) BrdU^+^ surface increase with loading. (*F*) Ps.Osx^+^ BrdU^+^ cells increase with loading. Data shown as mean ± SD (*n* = 9; males = 4, females = 5). * Signifies *p* < .05 by Student's *t* test. tdTomato = red; calcein = green; BrdU = white; 4,6‐diamidino‐2‐phenylindole (DAPI) = blue; Mu = muscle; Ps‐periosteum; B = bone.

In 5‐month‐old iOsx‐Ai9 mice, the periosteal surface of nonloaded tibias had negligible BrdU^+^ surface cells (<1%; Fig. 5 *A*, *E*), whereas loaded tibias had numerous BrdU^+^ cells including a mix of tdTomato^+^ cells at or near the bone surface, and tdTomato^−^ cells above the layer(s) of positive cells (Fig. 5 *B–D*). On the loaded tibia, 29% of the tdTomato^+^ surface adjacent to the bone surface was BrdU^+^ (Fig. 5 *E*). Similarly, of the tdTomato^+^ cells directly on the periosteal bone surface, there was a significantly greater fraction of BrdU^+^ cells in loaded compared with nonloaded tibias (31% and 1%, respectively, *p* = .002; Fig. 5 *F*). A trend for an increase of tdTomato^+^BrdU^+^ surface was found on the calcein surface in loaded compared with nonloaded tibias (12% and 0%, respectively; *p* = .052).

**Figure 6:**
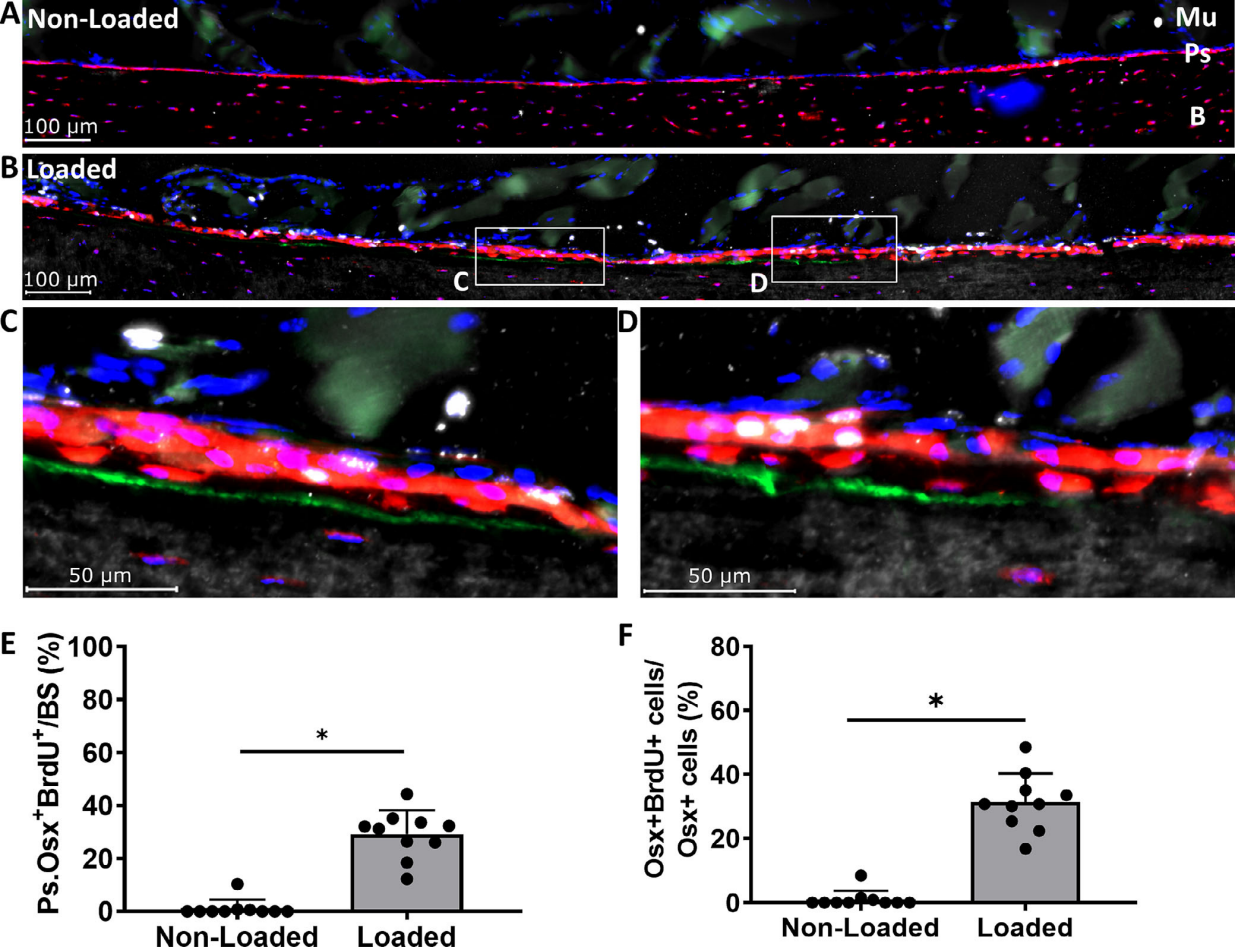
(*A, B*) Representative 5‐bromo‐2′‐deoxyuridine (BrdU) images of the region of interest from nonloaded and loaded tibias in 12‐month‐old mice. (*C, D*) 40× magnifications from loaded tibias. (*E*) Total Ps.Osx^+^ (periosteum osterix positive) BrdU ^+^ surface increase with loading. (*F*) Ps.Osx^+^ BrdU ^+^ cells increase with loading. Data shown as mean ± SD (*n* = 10; males = 6, females = 4). * Signifies *p* < .05 by Student's *t* test. tdTomato = red; calcein = green; BrdU = white; 4,6‐diamidino‐2‐phenylindole (DAPI) = blue; Mu = muscle; Ps = periosteum; B = bone.

Similar to the results at 5 months, nonloaded tibias of iOsx‐Ai9 mice at 12 months showed little to no BrdU^+^ periosteal surface (<2%; Fig. 6 *A*), whereas on loaded tibias, 29% of the tdTomato^+^ periosteal surface was BrdU^+^ (*p* < .0001; Fig. 6 *B–E*). Likewise, of the tdTomato^+^ cells directly on the periosteal bone surface, there was a significant increase in the number of BrdU^+^ cells in loaded compared with nonloaded tibias (31% and 1%, respectively, *p* < .0001; Fig. 6 *F*). Finally, a significant increase of tdTomato^+^BrdU^+^ surface was found on the calcein surface in loaded compared with nonloaded tibias (19% and 0%, respectively; *p* < .0001). Thus, proliferation of pre‐existing Osx‐lineage cells contributes to periosteal cells at sites of loading‐induced bone formation in 5‐ and 12‐month‐old mice.

## Discussion

Mechanical loading can induce a quiescent bone surface to a state of active mineral apposition. Some histological features of the cells on the bone surface in regions of loading‐induced bone formation have previously been described.^4^, ^5^, ^8^, ^9^ Our objective was to use modern lineage‐tracing methods to begin to better define the origin of cells on the bone surface after anabolic mechanical loading, including those that arise via cell proliferation.

We first wanted to demonstrate iOsx‐Ai9 mice would have an anabolic response to mechanical loading and investigate the specificity of the inducible reporter system. Loaded tibias from 5‐ and 12‐month‐old mice had a significant increase in periosteal bone formation indices, indicating that mice responded to the mechanical strains applied. Dynamic histomorphometry revealed tibias were loaded near the lamellar to woven bone threshold, as woven bone was present in some samples. However, only lamellar bone was present after 5 days of loading as indicated by the (initial) calcein label, whereas any woven bone showed only incorporation of the (second) Alizarin label. Thus, we chose to perform subsequent histological analysis of lineage and cell proliferation of the surface cells after 5 days of mechanical loading to reflect a process of lamellar bone formation. This was confirmed by day 5 frozen sections, which showed only linear calcein labels. We also found, in our analysis of frozen histology, no effect of tamoxifen on the periosteal loading response after 5 days of mechanical loading, indicating all mice had a similar anabolic response. We then compared iOsx‐Ai9 mice with and without tamoxifen, and found less than 5% of the periosteal cells were labeled in the absence of tamoxifen, but with tamoxifen, nearly the entire periosteal surface was labeled. Similarly, in the absence of tamoxifen approximately 50% of the osteocytes were labeled, but with tamoxifen, nearly all osteocytes were labeled. Although this demonstrates that the Cre is leaky, it is primarily localized to the cells within the cortical bone, not the cells on the surface of the bone. Therefore, because the tibial loading model causes peak bone formation on the compressive periosteal surface,^28^, ^30^, ^31^ where cells are labeled upon tamoxifen administration, this loading model and reporter system are appropriate tools to evaluate the cells responding to anabolic loads on the bone surface.

We next investigated the contribution of Osx‐lineage cells to the initial anabolic response after loading. The periosteal surface of nonloaded tibias from 5‐ and 12‐month‐old mice were lined with tdTomato^+^ cells, indicating that cells on the quiescent bone surface are from a pre‐existing Osx^+^ lineage. After 5 days of mechanical loading, the periosteal surface of loaded tibias from 5‐ and 12‐month‐old mice continued to be lined with tdTomato^+^ cells, including the bone forming surfaces. Because tamoxifen was only administered prior to mechanical loading and was not given continuously, this indicates that the pre‐existing Osx^+^‐lineage cells, or their progeny, are the cells responding to mechanical stimulus to form bone at this time. Less than 1% of the periosteal surfaces of loaded tibias were tdTomato^−^, indicating that cells are not being recruited from an earlier (pre‐Osx) progenitor population to sites of bone formation at the 5‐day time point. Thus, we conclude that differentiation of osteoblast progenitors is not required for the initial loading response. Our data support the previous observations that osteoblasts can be activated by mechanical loading to contribute to loading induced bone formation.^3^, ^5^, ^8^, ^9^

We then examined the prevalence of proliferating cells at the site of loading‐induced bone formation. We administered BrdU in the drinking water of 5‐ and 12‐month‐old iOsx‐Ai9 mice to label any cells that replicated during the 5 days of mechanical loading. Nonloaded tibias showed few BrdU^+^ cells on the periosteal surface, demonstrating a quiescent bone surface at both ages. However, with mechanical loading, we saw an increase in the number of cells expressing BrdU. Specifically, approximately 30% of the Osx‐lineage cells on the periosteal surface arose via proliferation in 5‐ and 12‐month‐old mice. This demonstrates that not only does cell proliferation occur on the periosteal bone surface, but it identifies these proliferating cells from the Osx lineage. Importantly, we found an increase in the total number of Osx^+^ surface cells with loading, suggesting the activation of cells with loading allowed for more cells on the bone surface to arise from proliferation. However, although the increase in proliferation could be explained by the increase in cellularity, we did not try to account for the fate of every pre‐existing cell and therefore cannot rule out that some of the original cells were lost through apoptosis. It is important to note that in these experiments we wanted to identify the overall amount of cell proliferation in response to mechanical loading. Therefore, we cannot specifically comment on when proliferation starts or when the cells proliferated. Nonetheless, to our knowledge this is the first demonstration that Osx‐lineage cells on the periosteal bone surface proliferate in adult mice.^11^, ^21^ Furthermore, though in agreement with Turner and colleagues demonstrating cell proliferation occurs following mechanical loading,^9^ these results elucidate the identity of cells that arise via proliferation from mechanical loading. Liu et al. have reported that mechanical loading of tibias with a cortical defect increases proliferation of early osteoprogenitors in the defect, but not Osx^+^ preosteoblasts,^34^ indicating that the effects of loading are different in bone repair versus intact conditions. Although proliferation was prevalent in our study, the majority of Osx^+^ cells were not BrdU^+^, demonstrating that pre‐existing osteoblasts or bone‐lining cells activate to contribute to bone formation.^3^, ^5^, ^8^, ^9^, ^35^ Thus, we conclude that proliferation and activation of Osx‐lineage cells contribute to loading‐induced bone formation.

We also observed a number of periosteal cells not on the bone surface that were BrdU^+^, but were not derived from the pre‐existing Osx‐lineage cells. We chose to focus on the bone surface cells in this study, and did not further characterize these proliferating cells above the surface. Histologically they appear to be mesenchymal progenitors from the outer (fibrous) layer of the periosteum, and/or cells derived from the muscle lying just above the periosteum. It remains unknown whether these cells differentiate to become osteoblasts on the bone surface and contribute to bone formation at a later time, or if the Osx‐lineage cells continue to be the only source of bone‐forming cells. Although the signaling mechanisms of load‐induced cell proliferation or activation remain unknown, it is well‐established that osteocytes are mechanosensor cells that play a role in the anabolic response to loading.^36^ Recent in vitro evidence indicates mechanically stimulated osteocytes secrete factors that stimulate osteoblast and mesenchymal progenitor cell migration and proliferation.^37^ Although our in vivo study did not investigate this phenomenon, it offers a possible mechanism for loading‐induced periosteal cell proliferation.

Several studies have demonstrated a reduced anabolic response to loading with aging,^4^, ^27^, ^38^ and one report implicated reduced cell proliferation as a factor.^4^ These studies loaded mice at forces that engender the same mechanical strain to compare the loading response across different ages. However, because our two age groups were not strain‐matched, we were unable to make direct comparisons between the 5‐ and 12‐month‐old mice. Nonetheless, we note that when 5‐ and 12‐month‐old mice were loaded to forces that stimulate a predominantly lamellar response in each, a fraction of Osx‐lineage cells arose via proliferation on the periosteal surface in both age groups. Additional work is needed to determine if there is any age‐related change in proliferation of the Osx‐lineage cells.

In this study, we included both male and female mice at both ages. Our dynamic histomorphometry data demonstrated that, although the sexes were loaded to different forces (and possibly different strains), they made similar amounts of bone. Importantly, we found sex had no effect on histological cellular outcomes related to lineage or proliferation. Therefore, we grouped the data by sex as there was no statistical difference in their cellular response under conditions when they were forming similar amounts of bone. However, because male and female mice were not strain‐matched, we cannot say whether there are sex‐dependent differences in the mechanical strain at which bone formation was induced in these iOsx‐Ai9 mice.

In summary, we performed in vivo mechanical loading of mouse tibias to evaluate the periosteal cellular response in a region of peak compressive strain. Our results demonstrate that in both 5‐ and 12‐month‐old iOsx‐Ai9 mice, pre‐existing cells of the Osx lineage cover the majority of surfaces where there is active loading‐induced bone formation. This indicates that the initial wave of new bone formation that occurs after mechanical loading is attributed to cells already committed to the Osx lineage, not the recruitment of earlier progenitor cells. We identified Osx‐lineage cells that arose via proliferation at sites of bone formation, as well as Osx‐lineage cells that had not proliferated. We conclude that both proliferation and activation of pre‐existing Osx‐lineage cells are essential features of the periosteal response to mechanical loading.

## Disclosures

The authors state they have no conflicts of interest.

## Supporting information


**Figure S1**: Supplementary Information.

**Figure S2**: Supplementary Information.

**Table S1** Supplementary Information.
