# Supplementary figures and images for: Proliferation and Activation of Osterix‐Lineage Cells Contribute to Loading‐Induced Periosteal Bone Formation in Mice
Source: JBMR Plus. 2019 Sep 11;3(11):e10227. doi: 10.1002/jbm4.10227 (PMC6874181; doi:10.1002/jbm4.10227)

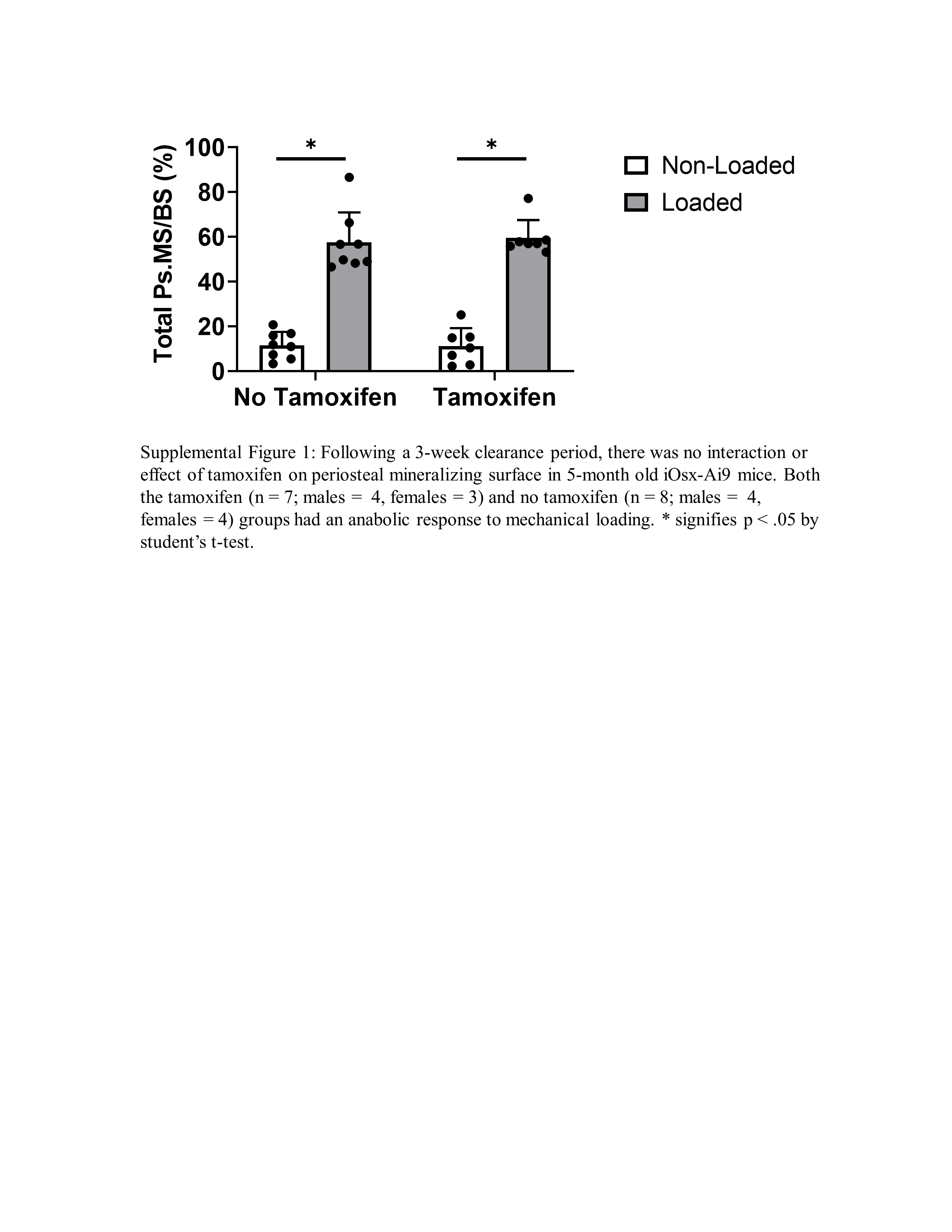

Supplement: Supplementary file 1 — Figure S1: Supplementary Information. [file JBM4-3-na-s001.tif]

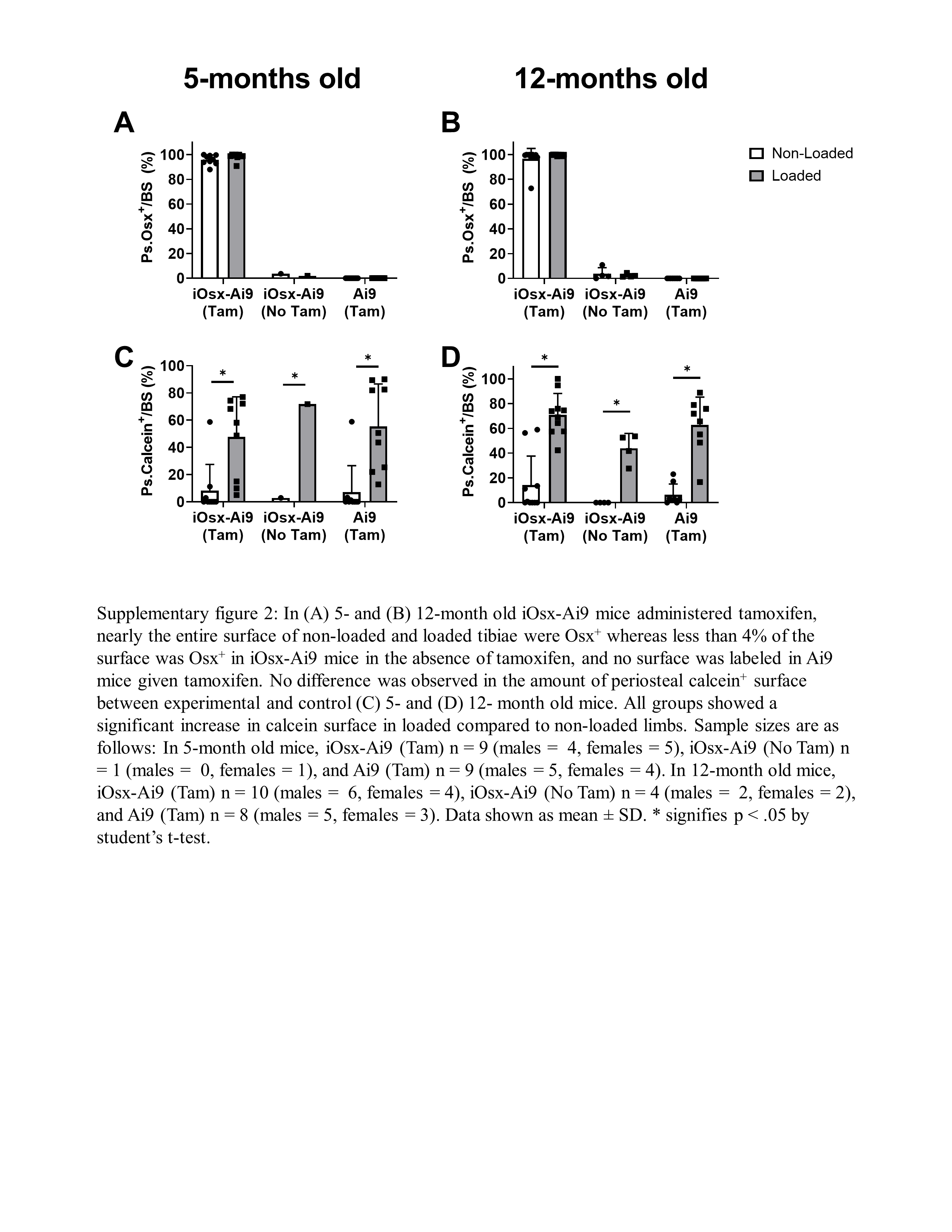

Supplement: Supplementary file 2 — Figure S2: Supplementary Information. [file JBM4-3-na-s002.tif]

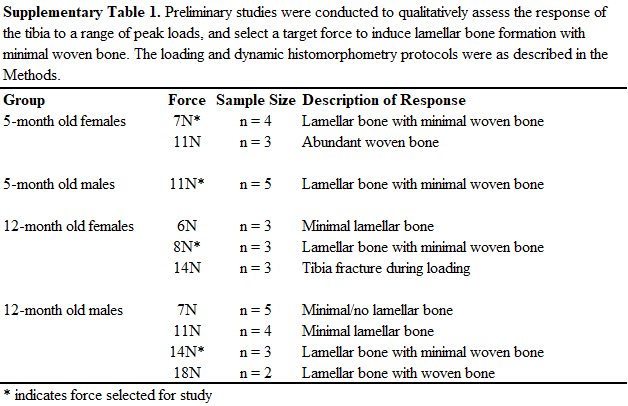

Supplement: Supplementary file 3 — Table S1 Supplementary Information. [file JBM4-3-na-s003.tif]
